# Monitoring of Cellular Changes in the Bone Marrow following PTH(1-34) Treatment of OVX Rats Using a Portable Stray-Field NMR Scanner

**DOI:** 10.1155/2017/7910432

**Published:** 2017-05-30

**Authors:** Inbar Hillel, Itzhak Binderman, Yifat Sarda, Uri Nevo

**Affiliations:** ^1^Department of Biomedical Engineering, Faculty of Engineering, Tel Aviv University, Ramat Aviv, 69978 Tel Aviv, Israel; ^2^Department of Oral Biology, School of Dental Medicine, Tel Aviv University, Ramat Aviv, 69978 Tel Aviv, Israel

## Abstract

Osteoporosis is characterized by reduction in trabecular bone in conjunction with increased marrow cell adiposity. While these changes occur within weeks, monitoring of treatment efficacy as performed by DEXA is sensitive only to long-term changes. MRI is sensitive to bone marrow changes but is less affordable. In a recent study, we have shown that a stray-field NMR can monitor bone marrow cellular changes that are related to osteoporosis.* Objectives. *To demonstrate sensitivity of a low-field tabletop NMR scanner to bone marrow dynamics following hormonal treatment in rats.* Methods. *Two-month-old female rats (*n* = 36) were ovariectomized (OVX) and dosed for the ensuing 3 or 5 weeks with 20 mg/kg of PTH(1-34). Hind limbs femurs and tibiae were isolated and underwent ex vivo microradiography and histology and NMR relaxometry at 6 weeks (preventive experiment) and 11 weeks (therapeutic treatment experiment) after OVX.* Results. *OVX rats developed osteoporotic changes including adipogenic marrow compared to Sham and PTH treated rats. *T*_2_ and ADC NMR relaxation coefficients were found to correlate with marrow composition.* Conclusions*. This study suggests that stray-field NMR, an affordable method that is sensitive to the rapid cellular changes in bone marrow, may have a clinical value in monitoring hormonal treatment for osteoporosis.

## 1. Introduction

### 1.1. Osteoporosis Treatments Targeting the Bone Marrow

Normally, therapeutic mechanisms to osteoporosis include the inhibition of bone resorption by inactivation of osteoclasts and the use of agents that promote bone mineralization. For more than 30 years, the use of aminobisphosphonates was the main treatment to inhibit osteoclast bone resorption activity without stimulating bone formation [1]. Other methods include estrogen hormone replacement, nutrition additives like calcium and vitamin D, and treatment with parathyroid analogues (PTH) [2]. Importantly, the latter operates by transforming the bone marrow population from adipocytic to osteoblastic and hematopoietic marrow [3]. Adipocytes and osteoblasts share common mesenchymal stem cells (MSCs) in the bone marrow, such that increased adipogenesis may be associated with decreased osteoblastogenesis [4]. Reduced osteoblast differentiation and normal function of osteoclasts are associated with increased marrow adipogenesis, leading to osteoporosis and disuse osteopenia. Thus, although osteoporosis has a variety of causes, a key mechanism is decreased osteogenic differentiation and increased adipogenic differentiation in the bone marrow [5, 6].

PTH not only exerts anabolic action by stimulating osteoblastic bone formation but also upregulates haematopoiesis by improving bone marrow microenvironment [7]. PTH(1-34), a recombinant human parathyroid hormone analogue containing the 34 residues, is currently used as an anabolic drug to treat osteoporosis in animals and humans [8] and to increase bone mass [9]. Importantly, PTH treatment is ineffective in many cases and chronic treatment over long periods (years) may have a catabolic effect [2]. It is therefore crucial to develop a bench top device for clinicians that is able to detect such early cellular changes at the different stages of the treatment. Such monitoring should preferably probe the bone marrow and not the bone mineral mass.

### 1.2. MRI Detection and Monitoring of Osteoporosis

Osteoporosis is commonly diagnosed by quantifying the BMD using dual energy X-ray absorptiometry (DEXA) [10]. Other methods include single-energy X-ray absorptiometry, single/dual photon absorptiometry, quantitative computerized tomography (QCT), and quantitative ultrasound [11]. It should be noted that these methods are effective when significant loss of bone is present. Over the last decade the use of nuclear magnetic resonance imaging (NMR and MRI) for the study of osteoporosis has expanded, in both preclinical and clinical studies with developments of 2D or 3D high-resolution MRI that enable the study of trabecular bone [12, 13], thus probing the cortical bone mass like the DEXA and QCT. However, MR can also track the rapid cellular changes in the bone marrow following treatment, by acquiring relaxometry coefficients.

The relaxation coefficients are the primary components that describe an NMR signal. The primary coefficients are *T*_1_, the spin-lattice relaxation that measures the decay of the NMR signal due to return to thermal equilibrium, and *T*_2_ and *T*_2_^*∗*^, the spin-spin relaxation time and the inverse of field inhomogeneity, respectively. Diffusion is also highly indicative of the composition and the viscosity of the tissue (both the intra- and extracellular compartments) and of the barriers that microstructures in the tissue impose on the displacement of water molecules. Diffusion is typically quantified by the apparent diffusion coefficient (ADC) that combines these multiple factors that affect water displacement in the tissue. Maris et al. [14] have postulated the assumption that, in addition to the known effect of trabecular bone loss, the presence of fat in osteoporotic marrow modulates MR relaxation times by an increase in *T*_2_ and *T*_2_^*∗*^ and by the decrease in *T*_1_. *T*_2_^*∗*^ and *T*_2_ were found to be the best discriminators of patients with osteoporosis from control subjects. Wehrli et al. [15] reported, for example, typical values of 15–25 sec^−1^ for *R*_2_ in the marrow of the lumbar spine and femoral neck of adults. These values had a negative correlation with the marrow fat content, with correlations of *r* = 0.46 and *r* = 0.82, respectively. Griffith et al. [16] measured the lumbar ADC but found that it did not change significantly with bone density. However, a mild correlation indicated that the marrow ADC decreases as fat content increases. Yeung et al. [17] demonstrated vertebral diffusion restriction in patients with osteoporosis. This reduction was attributed to the increased fat packing of bone marrow accompanying a reduction in bone density as a consequence of osteoporosis. Mean ADC values in this study significantly dropped from 0.49 · 10^−5^ cm^2^/sec for healthy women to 0.41 · 10^−5^ cm^2^/sec in postmenopausal women with a reduced BMD (group separated into three subgroups with osteopenia, osteoporosis, and osteoporosis with fractures, with mean *T*-scores of −1.75, −3.19, and −2.8, resp.).

### 1.3. Low-Stray-Field NMR as a New Modality for Monitoring Osteoporosis

The above studies were all done with high-field MRI scanners and demonstrate the sensitivity of MRI to osteoporosis and specifically its value when bone marrow composition is probed. However, high-field MRI is not yet a standard measurement for detection of osteoporosis, in spite of its clinical value, mainly because of its high cost. Unilateral NMR devices were developed for the last three decades for applications in nondestructive testing, offering mobility, open geometry, and low costs [18]. It should be noted that their open geometry along with the small magnet size limits these devices to being typically characterized by a low magnetic field strength (0.1–0.3 T) that is also highly inhomogeneous (stray gradient of ~7–15 T/m). The latter is the main drawback that results in a very low sensitivity.

In spite of these drawbacks, a few works already examined the use of unilateral scanners for examination of tissues [19–24] and even in a clinical study that demonstrated the application of examination of skin aging with a stray-filed NMR scanner [25]. Sarda et al. [26] have shown that a stray-field NMR can in principle be used to monitor osteoporosis related changes to the bone marrow. They have shown significant changes in the central zone of the bone marrow metaphysis (metaphysis that included only bone marrow spaces) of ovariectomized (OVX) rats, relative to Sham operated rats. Bone marrow adiposity was detected by reduction in *T*_1_ relaxation time, reduction in ADC, and an increase in *T*_2_ in OVX bones, 3-4 months after operation.

This study explored the feasibility of using an inhomogeneous portable NMR device as a potential modality to track the treatment of osteoporosis. We report the monitoring of PTH treatment efficacy, when given as a preventive drug and as a treatment for severe osteoporosis in an animal model. We suggest that the stray-field NMR can potentially be used to monitor treatment efficacy in relatively short times, by probing the bone marrow content in early stage rather than probing the end-point of bone loss).

## 2. Methods

### 2.1. Animals and Care

The research was carried out in accordance with the Helsinki Declaration at the animal care unit of the Faculty of Medicine, Tel Aviv University, M-15-017. Female Sprague Dawley rats (*n* = 36, 2 months old) underwent a bilateral ovariectomy (OVX group) or a superficial dorsal midline incision (Sham/control group). During the operations, rats were anesthetized with Ketamine and Xylazine (0.25/0.1 cc) and received Rimadyl (0.2 cc) as an analgesic and Baytril (5 mg/kg) as antibiotics.

OVX rats were further split into three groups: a group undergoing a preventive treatment with human PTH (1-34) (prevention group), a group treated with PTH after osteopenia was developed (therapeutic treatment group), and a nontreated group. Prevention group received daily injections of vehicle or PTH (1-34) (human) subcutaneously, at a dose of 20 *μ*g/kg for five weeks, starting one week after operation. Therapeutic treatment group rats were permitted to lose bone for 8 weeks to establish severe osteoporosis before the initiation of treatments, which lasted for three weeks. Postoperation, rats were housed in groups of 2-3 animals per cage, with free access to a standard rat diet and water; they were weighed weekly and checked for postoperative abnormalities.

Prevention group was sacrificed 6 weeks after operation by an overdose of CO_2_ and the femur and tibia bones from the hind legs were isolated for ex vivo scans. Discriminating different soft tissues, one from the other, is not feasible with the current stray-field NMR devices, whereas, biological imaging with stray-field scanners is impractical with respect to the duration of the scan [27]. For that reason, in this study we isolated the bones from other soft tissues.

**Figure 1:**
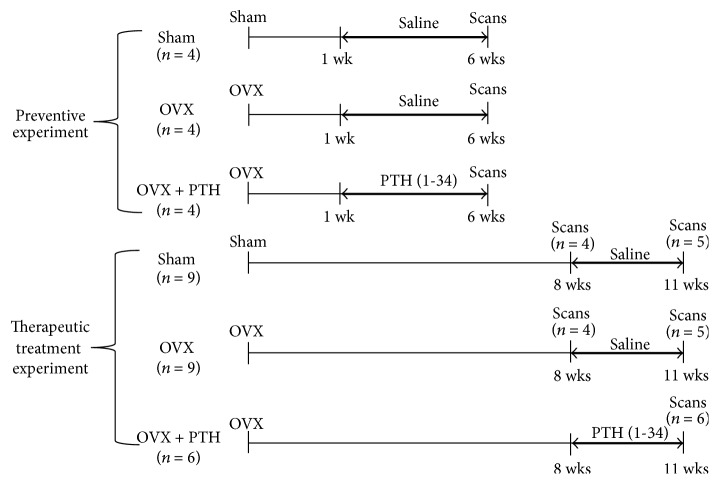
Line diagrams showing experimental design for PTH treatment of ovariectomized rats. The time after operation is shown below the line and the type of treatment (PTH or saline) is shown above the bold arrow. In experiment 1 (preventive experiment), 1 week after operation the rats received daily injections during 5 weeks whereas in experiment 2 (therapeutic treatment experiment) rats were permitted to lose bone for 8 weeks before the initiation of treatments and were treated for 3 weeks.

Therapeutic treatment group rats were sacrificed at two points: 8 and 11 weeks after operation. Sham and nontreated rats were sacrificed at all the three time points. In practice, since the therapeutic treatment and the prevention groups are noncomparable, the groups were analyzed and compared as two separate experiments: one studying the capability of detecting a preventive scenario and another studying capability of monitorign the recovery of a bone marrow (see Figure 1).

Bones were kept in a moist petri dish with phosphate buffered saline and preserved at a temperature of 4°C, for no more than 8 hours after their extraction. The bone marrow cavity of the proximal diaphysis edge of both selected bones was scanned. The NMR scans were performed on the same day the rats were sacrificed. Later, half of the bones were preserved in a refrigerator for microradiography measurement and half in formalin (4%) for histology. It should be clarified that while the exact time course of the treatment and the time-point of sacrifice were different, the postmortem treatment of the bones, the NMR protocol, and the analysis were kept the same.

### 2.2. Hardware

NMR scans were performed using the NMR-MOUSE (Mobile Universal Surface Explorer, Magritek, NZ). The device consists of a permanent U-shaped magnet, resulting in *B*_0_ = 0.3 T (with a resonance frequency of ~13.6 MHz) with a highly flat sensitive volume at a distance of 25 mm parallel to the magnet's surface (*XY* plane) and with a strong stray-field gradient of ~8 T/m. A 3 mm surface RF coil was used for transmission and reception. Because the gradient causes an inhomogeneity for both magnetic fields, *B*_0_ and *B*_1_, the signal-to-noise ratio (SNR) of our measurement was very low. Since the echo time (TE) that can be used should (usually) be much shorter than *T*_2_ of the sample, of the order of 60 microseconds, Carr-Purcell-Meiboom-Gill (CPMG) sequence with multiecho train can be applied in every encoding step. By summing the generated echoes together, the total amplitude of the detected signal can be significantly enhanced, as demonstrated before by Bluemich et al. [18]. The pulse sequences are tailored specifically to optimize a scan with an inhomogeneous magnetic field.

### 2.3. Bones Dimension and Weights

Femurs and tibiae were removed and their dimensions and weights were measured. Bones lengths and diameters were measured and were weighted. Each measurement was repeated three times for statistical purposes.  Table 1 summarizes the measured data for the prevention and therapeutic treatment experiments, respectively.

### 2.4. Histology

The histology and microradiography were aimed at showing qualitative support of the effectiveness of PTH on bone deposition and changes in the bone marrow. We therefore used only a limited amount of samples for this purpose.

Femurs and tibiae were removed and dissected free of soft tissues. The bones were fixed in 4% formalin and processed by conventional paraffin embedding. The sections were taken from the relevant scanned area in the bones, at the diaphysis proximal edge. The slices were stained with hematoxylin and eosin and imaged with AE31 inverted microscope (Motic, Xiamen, China), magnification ×4/×10.

### 2.5. Microradiography

Femurs and tibiae were removed and dissected free of soft tissues. Microradiography images were conducted over the bone samples with the FAXITRON cabinet X-ray system (Hewlett-Packard, Palo Alto, CA) [26, 28]. Each slide was then imaged with an LG camera (13 Megapixels) fixed with a camera bracket. Preprocessing of the grey-scale images was performed by normalization of intensities (to a 0-1 scale) followed by binary digitization (threshold at 0.5), (MATLAB, The Mathworks, Natick, MA).

### 2.6. NMR Scan Protocol

*Bone Handling*. Femurs and tibiae were removed and dissected free of soft tissue. The vitality of the bones was maintained by putting each bone in its own petri dish, which was moistened with phosphate buffered saline and preserved at a temperature of 4°C.

Bones were covered tightly with plastic wrap, to prevent dehydration and potential degradation while scanning. Each bone was placed with chosen area of the proximal metaphysis above the center of the RF surface coil. Metaphysis is the wider part at the end of the diaphysis shaft, where active growth of a long bone occurs, and is richly supplied with blood [29].

*Profiling.* An NMR 1D profiling in the longitudinal direction was acquired along the radial axis of the bone, for definition of the scanning zones (either the center or periphery of the marrow cavity).

**Figure 2:**
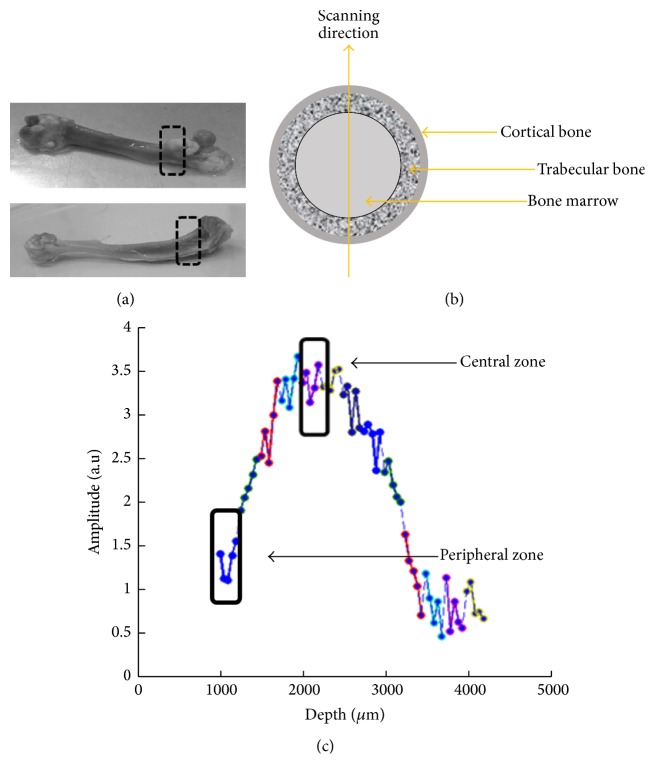
Scanning zones. (a) Scanning regions in proximal tibia and femur metaphysis. (b) Scanning direction in bone cross-section illustration. (c) 3.25 mm depth profile of the proximal tibia diaphysis. Data from different depth scans are marked by different colors. The chosen regions for *T*_1_, *T*_2_, and ADC measurements are marked at either the center or periphery zones of the metaphysis.

As mentioned before, the peripheral zone is considered to be more sensitive to the cellular changes; however, the central zone has the advantage of allowing a higher SNR. Excitation of different depths (with the same resonance frequency) was performed by a lift-controlled upward shift of the magnet and the coil along the *z*-direction; the lift position was defined relative to the fixed sample. The configuration and the applied protocol resulted in a penetration depth of approximately 3.25 mm. Since the SNR of each depth in our measurements drops significantly away from its center, only a 250 *μ*m image segment was reconstructed for each excited slice (data from each lift position is colored differently as shown in Figure 2). We set 13 lift steps with the following acquisition parameters: TR = 1000 ms, 24 averages (24 sec for depth acquisition), 700 echoes, TE = 100 *μ*s, 120 sampling points, and 0.5 *μ*s dwell time (50 *μ*m spatial resolution). After the desired profile was obtained, the bone marrow metaphysis center (metaphysis that included only bone marrow spaces) or periphery (metaphysis that included trabeculae and bone marrow spaces) was set for elaborated *T*_1_, *T*_2_, and diffusion scans.

*T*_2_ *Measurements*. *T*_2_ was acquired using a CPMG-like sequence with 200 averages, 700 echoes, TR = 1800 ms, TE = 65 *μ*s, and scan time ~6 min. An estimation method based on statistical signal processing was applied to the acquired CPMG train [30].

*T*_1_ *Measurements*. *T*_1_ was acquired using a saturation recovery pulse sequence, combined with a CPMG train after the main encoding period. We used 18 different recovery times spaced exponentially between 0 and 2200 ms with 36 averages, 350 echoes, TR = 2500 ms, TE = 65 *μ*s, and scan time ~27 min.

*Diffusion Measurements*. ADC was acquired using a static gradient stimulated echo [31] with 14 different *B*-values between 50 and 1000 s/*μ*m^2^, 200 averages, 250 echoes, TR = 400 ms, TE = 65 *μ*s, and scan time ~19 min. Again, a CPMG train was added to improve our SNR.

### 2.7. Statistical Analysis

A one-way Kruskal-Wallis test was used as a nonparametric test for the comparison of independent variables of each group (OVX, OVX + PTH, and Sham) for tibiae and femurs bones separately. In addition, a post hoc multiple-comparison analysis was used with Bonferroni method. Values of *P* < 0.05 were considered statistically significant. The NMR results are presented as box plots, the lines represent the lower quartile, median, and upper quartile values, and the notches in boxes graphically show the 95% confidence interval about the median of each distribution.

## 3. Results

Animals and their long bones average weights of both prevention and therapeutic treatment experiments are presented in Table 1. In the prevention experiment, a significant increase in the mean weight of OVX rats in comparison to Sham rats was measured after 6 weeks (*P* < 0.007). No significant changes in the animal's weights were measured at 6 weeks between PTH treated rats (PTH + OVX) and OVX rats. In contrast to effects of PTH + OVX on weight of rats, tibiae average weight of OVX + PTH increased compared to Sham group (*P* < 0.05) and when compared to OVX group (*P* = 0.064). No significant changes were measured of the weight, length, or diameter of femurs bones. In the therapeutic treatment experiment, a significant increase in the mean weights OVX and OVX + PTH rats compared to the Sham control mean weight (*P* < 5 · 10^−5^) was observed 11 weeks after operation. No significant changes in the animal's weights were measured between OVX and OVX + PTH groups. In this experiment, only femurs mean weight significantly increased in the OVX + PTH compared to OVX femurs mean weight (*P* < 0.05). No significant changes were measured in the tibiae bones average weight, length, or diameter between the three groups.

**Figure 3:**
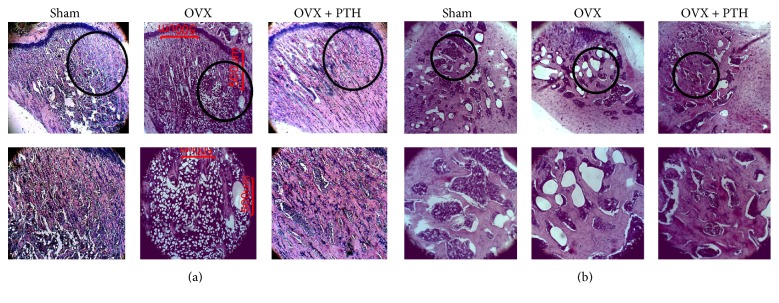
Prevention experiment reference measurements: examples of bone marrow cellularity histology (hematoxylin and eosin stain) of Sham, OVX, and OVX + PTH, treated with PTH at 6 weeks after operation: (a) bone marrow cellularity histology of tibiae bones; (b) bone marrow cellularity histology of femurs bones. OVX revealed a very high number of adipocytes and less dense trabecular bone compared with the Sham and PTH treated rats. Slides were visualized by Motic AE31 inverted microscope, magnification ×4/×10.

**Figure 4:**
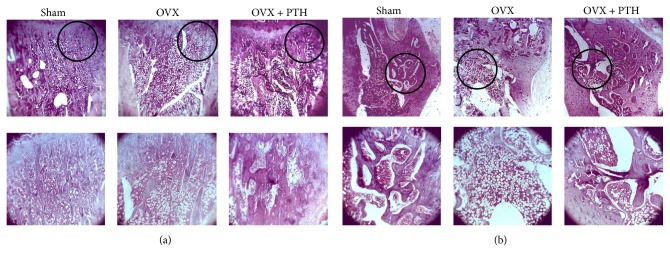
Therapeutic treatment experiment: examples of bone marrow cellularity histology (hematoxylin and eosin stain) of Sham, OVX, and OVX + PTH (a) tibiae and (b) femurs bones at 11 weeks after operation. OVX revealed a significantly greater amount of adipocytes compared with the Sham and PTH treated rats. Slides were visualized by Motic AE31 inverted microscope, magnification ×4/×10.

Qualitative histological observations of the bone marrow in the metaphysis (hematoxylin and eosin stain) of Sham, OVX, and OVX + PTH, treated with PTH at 6 weeks after operation (prevention experiment) and 11 weeks after operation (therapeutic experiment) are presented in Figures 3 and 4, respectively. The metaphysis of OVX (a) tibiae bones and (b) femurs bones is characterized by narrow trabeculae and low trabecular bone surface, while most of marrow spaces are filled with adipose tissue. In the PTH treated animals (PTH + OVX) pronounced hematopoietic marrow and wide trabeculae were present in metaphysis, like in Sham rats, in both experiments. The full set of microradiograph and histology figures appears in the supplementary material (in Supplementary Material available online at https://doi.org/10.1155/2017/7910432).

**Figure 5:**
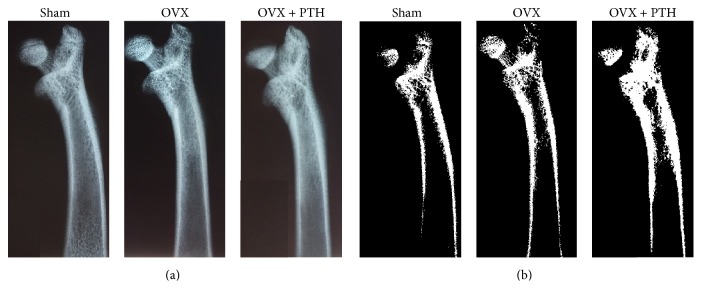
Prevention experiment reference measurements: example of microradiography of Sham, OVX, and OVX + PTH, treated with PTH at 6 weeks after operation. (a) Representative radiographs of femurs bones. (b) Radiographs following threshold analysis. PTH treatment increased the trabecular bone when compared with the Sham and the OVX controls. Slides were conducted with FAXITRON (a cabinet X-ray system, Hewlett-Packard).

**Figure 6:**
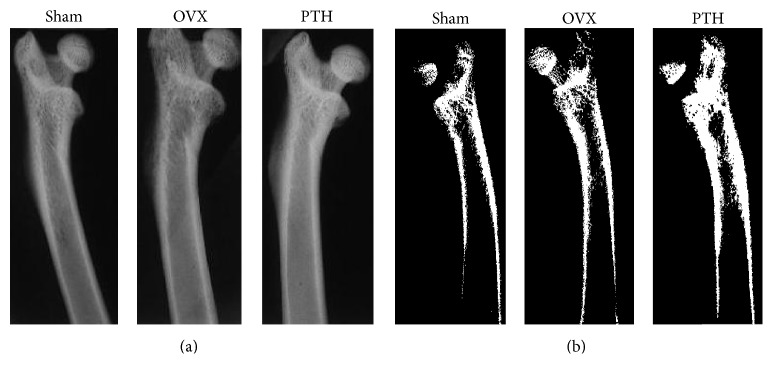
Therapeutic treatment experiment: example of microradiography of Sham control, OVX and OVX treated with PTH femurs bones at 11-weeks postoperation. (a) Representative radiographs (b) Radiographs following threshold analysis. PTH treatment increased the trabecular bone compare with the Sham and the OVX controls. Slides were conducted with FAXITRON (a cabinet X-ray system, Hewlett-Packard).

In addition, depictive microradiography images of Sham, OVX, and OVX + PTH, treated with PTH at 6 weeks after operation (prevention experiment) and 11 weeks after operation (therapeutic experiment) are shown in Figures 5(a) and 6(a), respectively. In the prevention experiment, threshold analysis emphasizes the increase in width of trabecular bone, after 5 weeks of PTH treatment of OVX rats (Figure 5(b)). Similar results were observed in the treatment efficacy experiment (Figure 6(b)).

**Figure 7:**
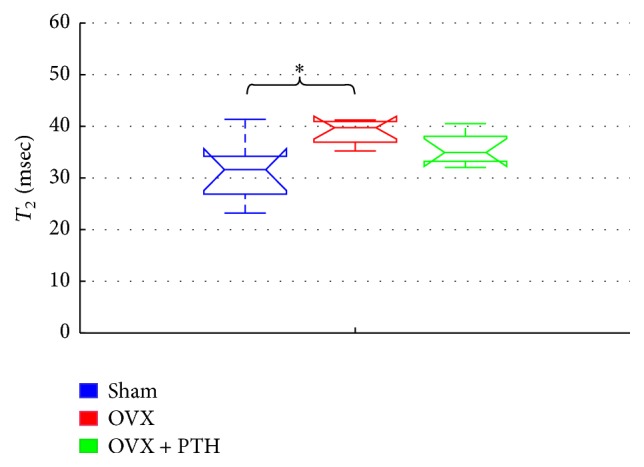
Prevention experiment – *T*_2_ measurements 6-weeks postoperation, measured at peripheral zone of metaphysis of femurs bones. Significant increase of *T*_2_ for the femurs of OVX bones compared to the Sham operated control group at peripheral zone of metaphysis (by Kruskal-Wallis test followed by Bonferroni multiple-comparison test ^*∗*^*P* < 0.008).

Figure 7 displays *T*_2_ relaxation time derived from femurs bones that were assessed six weeks after operation, at the peripheral zone of metaphysis that included trabeculae and bone marrow spaces. Significant increase was found in *T*_2_ of the OVX femurs bones compared to Sham operated control bones [OVX *T*_2_: 35–41 (ms); Sham *T*_2_: 23–41 (ms); *P* = 0.0071]. Difference in *T*_2_ values of the PTH treated group (outlined in the OVX-PTH bar) compared to the other groups is statistically insignificant. Nevertheless, median and percentile values suggest that there is a shift of *T*_2_ values of the PTH treated group towards *T*_2_ values of normal (Sham) bones [OVX + PTH *T*_2_: 32–40 (ms)]. Since, in the present experiment the PTH treatment started 1 week after OVX, we found that *T*_2_ and other NMR parameters did not differ significantly from these of the Sham group, possibly implying the effectiveness of PTH to prevent cellular changes toward osteoporosis.

**Figure 8:**
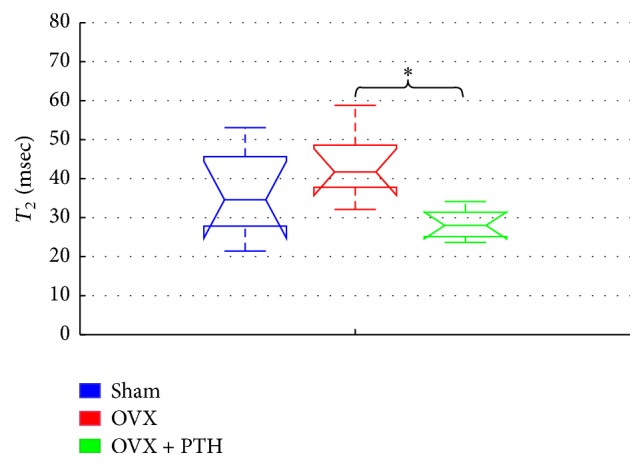
Prevention experiment: *T*_2_ measurements 6 weeks after operation, measured at peripheral zone of metaphysis of tibiae bones. Significant increase for the tibiae OVX bones compared to the OVX treated with PTH when measured in the peripheral zone of the bone marrow cavity (by Kruskal-Wallis test followed by Bonferroni multiple-comparison test ^*∗*^*P* < 0.006).

In addition, we found a significant increase in *T*_2_ values of tibiae OVX bones compared to the OVX treated with PTH [OVX *T*_2_: 32–58 (ms); OVX + PTH *T*_2_: 23–34 (ms); *P* = 0.0056] that were assessed six weeks after operation, at the peripheral zone of metaphysis (Figure 8). However, *T*_2_ of Sham tibiae bones was lower, yet not significantly lower than OVX tibiae bone, due to high SD and small amount of bones [Sham *T*_2_: 21–53 (ms)]. *T*_1_ and ADC measurements in the prevention experiment were taken in the central zone of the bone marrow cavity and showed general decrease in the OVX bones compared to the Sham and OVX + PTH groups (see Supplementary ‎1. 1.).

**Figure 9:**
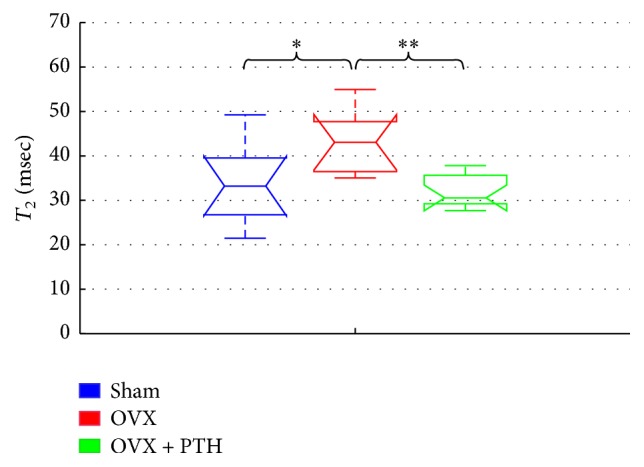
Therapeutic treatment experiment: *T*_2_ measurements of Sham, OVX, and OVX + PTH femurs bones, measured 11 weeks after operation. Significant increase in *T*_2_ for the femurs OVX bones compared to the OVX + PTH and the Sham femurs bones when measured in the peripheral zone (by Kruskal-Wallis test followed by Bonferroni multiple-comparison test ^*∗*^*P* < 0.04, ^*∗∗*^*P* < 0.02).

Figure 9 the displays *T*_2_ relaxation time derived from femurs bones measured eleven weeks after operation and 3 weeks of PTH treatment (OVX + PTH). A significant increase in *T*_2_ of OVX group (OVX *T*_2_: 35–53 (ms)) than Sham (Sham *T*_2_: 30–42 (ms)) and OVX + PTH femur bones (OVX + PTH *T*_2_: 28–37 (ms)), [*P* < 0.04 (Sham Versus OVX), and *P* < 0.02 (OVX + PTH versus OVX)] was measured at the peripheral zone metaphysis that included trabeculae and bone marrow spaces.

**Figure 10:**
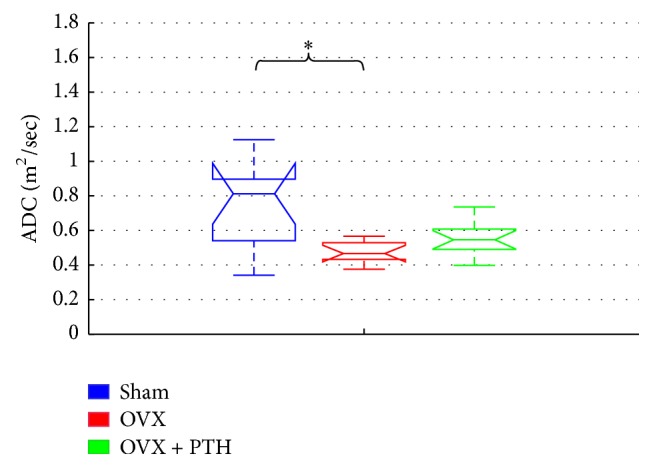
Therapeutic treatment experiment: ADC measurements of Sham, OVX, and OVX + PTH tibiae bones, measured 11 weeks after operation. Significant decrease for the tibiae OVX bones compared to the Sham tibiae bones when measured in the central zone (by Kruskal-Wallis test followed by Bonferroni multiple-comparison test ^*∗*^*P* < 0.006).

Figure 10 displays the ADC parameter derived from tibiae bones measured eleven weeks after operation and 3 weeks of PTH treatment (OVX + PTH). A significant decrease in the ADC for the OVX group (OVX ADC: 0.37-0.55E-9 (m^2^/s)) was found compared to the Sham group (Sham ADC: 0.34-1.12E-9 (m^2^/s)) [*P* < 0.006 (Sham versus OVX)] when measured at the central zone of metaphysis that included only the bone marrow spaces. While the ADC of the OVX + PTH group was in general higher than OVX group and close to Sham group (OVX + PTH ADC: 0.39-0.73E-9 (m^2^/s)), no statistically significant difference was found. It seems at this point that the *T*_2_ parameter is more sensitive than *T*_1_ and ADC to cellular bone marrow changes in response to hormone treatment (PTH) and changes due to OVX. Additional NMR results of *T*_2_, *T*_1_, and ADC are presented in Supplementary ‎1.2.

## 4. Discussion

This study uses an animal model to provide a proof-of-concept that the detection of treatment efficacy in osteoporosis may be feasible using a portable low-stray-field NMR device. *T*_2_ and the ADC change significantly due to ovariectomy and were then influenced by PTH treatments, in both a preventive experiment and in a therapeutic treatment experiment. It should be noted that in the therapeutic treatment experiment only 3 weeks of PTH was given to the OVX rats, while 5 weeks of PTH was given in the preventive experiment. In both experiments, *T*_2_ relaxation time increased as the disease evolved. This result is in line with Wehrli et al. which showed that *R*_2_ (1/*T*_2_) is inversely proportional to the percentage of fat in the bone marrow [15]. Sarda et al. also found a significant increase in *T*_2_ for OVX bones in comparison to Sham controls, when measured at the central zone of bone marrow cavity, from 14 weeks and onward [26]. Here, in the preventive experiment, a significant increase in *T*_2_ of the OVX bones was found as early as 6 weeks after operation, when measured at the peripheral zone of the metaphysis (a region reach of trabecular bone that interfaces with marrow cells). In the same experiment, PTH(1-34) treatment for 5 weeks did reverse the trend of *T*_2_ of OVX in tibiae and femurs bones (though being found significant only in the tibiae). We therefore suggest that the NMR device is sufficiently sensitive to detect early cellular changes in the marrow both after OVX and following preventive treatment with PTH. The NMR results are in agreement with bone parameters in the metaphysis of the present study.

Today, the effectiveness of anabolic drugs, like PTH, in therapeutic treatment of bone is monitored by DEXA test, with a typical lag of 1-2 years between measurements, to obtain a sufficient significance, upon recovery of the BMD. In contrast, if cellular changes from adipocytic towards osteoblastic and hematopoietic bone marrow were to be monitored clinically, an indication of an anabolic therapeutic effect could be obtained by 1–4 months [32]. This suggested application extends to all other types of treatments that are expected to affect marrow content, such as changes in endocrine, nutrition, or life style. On the contrary, this method is not expected to be sensitive to the treatment of osteoporosis by bisphosphonates that exhibits significant antiresorptive effect by suppressing osteoclasts function [33]. Thus, a clinically affordable method that is sensitive to the rapid changes following hormonal treatment may have a crucial effect on the management of the disease. It is typically assumed that PTH treatment should not extend beyond 6 months, which is the typical period for bone healing [34]. However, treatment duration that will result in optimal bone regeneration with minimal adverse outcomes and patient discomfort is unsolved and probably varies across patients [35]. A proposed stray-field NMR device that is sensitive to marrow cell changes can lead to optimization of PTH therapy timing duration and dose.

Finally, in this ex vivo measurements, the scanned region had a volume of 3 × 3 × 0.1 mm^3^. This led to a low signal-to-noise ratio (and demanded long scan times). In a clinical scenario, by improving hardware and software of future device, the typical volume of a scanned region of bone marrow can be in the order of 1 cm^3^ (~×1000 increase in volume relative to this study), with an obvious increase in sensitivity. The gradient may further be decreased at a given surface area by incorporating additional shim magnets [19]. Thus, if a clinical study will confirm these preclinical results, the stray-field NMR technology may provide a reliable means for monitoring anabolic treatments of osteoporosis.

## Supplementary Material

Supplementary 1. 1. provides additional NMR, histology and radiographs results from the prevention experiment. Supplementary 1. 2. provides additional NMR, histology and radiographs results from the therapeutic treatment experiment.

## Figures and Tables

**Table 1:** Animals and bones average weights in the prevention experiment (6 weeks after operation) and in the therapeutic treatment experiment (11 weeks after operation).

Experiment	Group	Animals weights (gr)	*n* femurs	Femurs weight (gr)	*n* tibiae	Tibiae weight (gr)
Prevention	SHAM	266.8 ± 5.8	8	0.85 ± 0.12	8	0.58 ± 0.16
	OVX	326.2 ± 17.7^*∗*^	8	0.84 ± 0.11	8	0.60 ± 0.08
	OVX + PTH	313.2 ± 46.1	8	0.90 ± 0.08	8	0.69 ± 0.07^*∗∗*^

Therapeutic treatment	SHAM	295.1 ± 18.2	10	0.99 ± 0.08	10	0.71 ± 0.06
	OVX	356.3 ± 16.4^*∗∗∗*^	10	0.97 ± 0.07	10	0.70 ± 0.08
	OVX + PTH	376 ± 7^*∗∗∗*^	12	1.10 ± 0.07^*∗∗*^	12	0.78 ± 0.08

Sham: Sham operated control rats treated with saline; OVX: ovariectomized rats treated with saline; OVX + PTH: PTH-treated ovariectomized rats. In the prevention experiment, a significant increase in the mean weight of OVX rats was found after 6 weeks, compared to the Sham control mean weight (^*∗*^*P* < 0.007). No significant changes in the animal's weights were measured at 6 weeks between OVX and OVX + PTH groups. Increase of the OVX + PTH tibiae average weight compared to Sham group (^*∗∗*^*P* < 0.05) and compared to OVX group (*P* = 0.064) was found. No significant changes were measured of the average weight of the femurs bones. In the therapeutic treatment experiment, a significant increase in the OVX mean animal weights compared to the Sham control mean weight (^*∗∗∗*^*P* < ****5 · 10^−5^) was observed 11 weeks after operation. No significant changes in the animal's weights were measured between OVX and OVX + PTH groups. In this experiment, only femurs mean weight significantly increased in the OVX + PTH compared to OVX femurs mean weight (^*∗∗*^*P* < 0.05). No significant changes were measured in the tibiae bones average weight between the three groups.
