# Supplementary material for: Monitoring of Cellular Changes in the Bone Marrow following PTH(1-34) Treatment of OVX Rats Using a Portable Stray-Field NMR Scanner
Source: J Osteoporos. 2017 May 30;2017:7910432. doi: 10.1155/2017/7910432 (PMC5468560; doi:10.1155/2017/7910432)
Supplement: Supplementary file 1 — Supplementary 1. 1. provides additional NMR, histology and radiographs results from the prevention experiment. Supplementary 1. 2. provides additional NMR, histology and radiographs results from the therapeutic treatment experiment. [file 7910432.f1.pdf]

1. Supplementary

1. 1. Prevention Experiment Additional Results

Figure 1 presents the NMR measurements of tibiae and femurs bones, which were measured 6 weeks post operation at the central zone of bone marrow cavity. T_2 measurements of OVX+PTH bones showed general decrease compare to OVX bones, though not significant. T_1 and ADC measurement in the central zone of bone marrow cavity were not repeatable and showed no one general trend for tibiae and femurs bones.

The full set of histological observations of the bone marrow and of SHAM, OVX and OVX+PTH bones, treated with PTH at 6-weeks post operation (prevention experiment) are presented in Figure 2. Additional depictive microradiography images of tibiae bones are presented in Figure 3. The metaphysis of OVX tibiae bones is characterized by narrow trabeculli and low trabecular bone surface, while most of marrow spaces are filled with adipose tissue. In the PTH treated animals (PTH+OVX) pronounced hematopoietic marrow and wide trabeculii were present in metaphysis, like in SHAM rats.

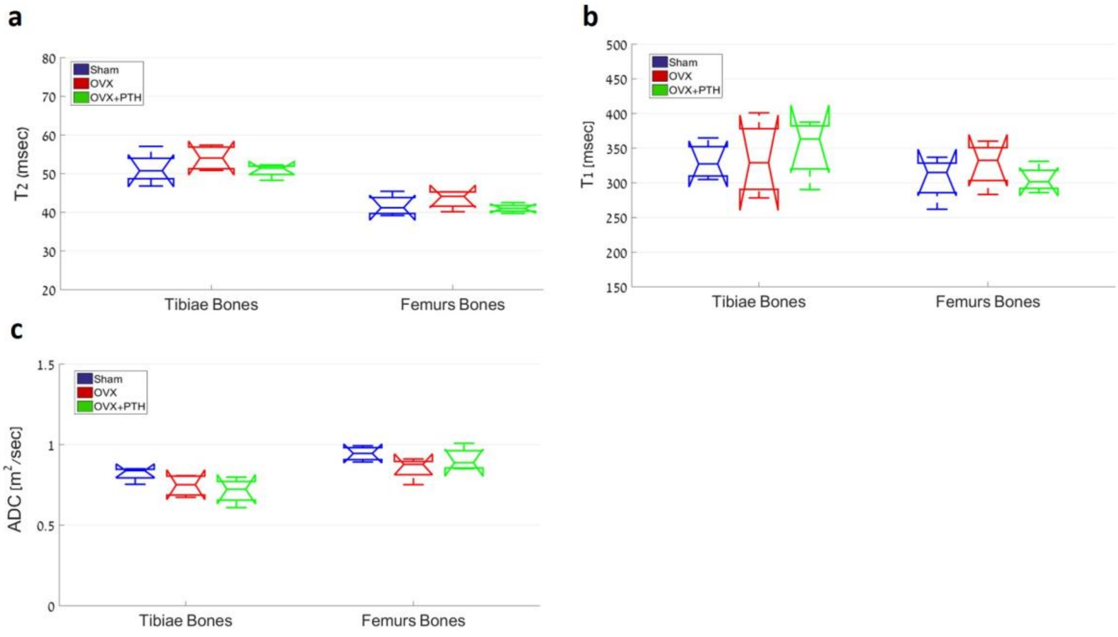

Figure 1: Prevention experiment – NMR parameters of tibiae and femurs bones measured at central zone of bone marrow cavity, 6-weeks post ovariectomy. (a) T_2 measurements of SHAM, OVX and OVX+PTH tibiae and femurs bones (b) T_1 measurements of SHAM, OVX and OVX+PTH tibiae and femurs bones (c) ADC measurements of SHAM, OVX and OVX+PTH tibiae and femurs bones. No significant changes were measured between the groups when measured in the central zone of bone marrow cavity.

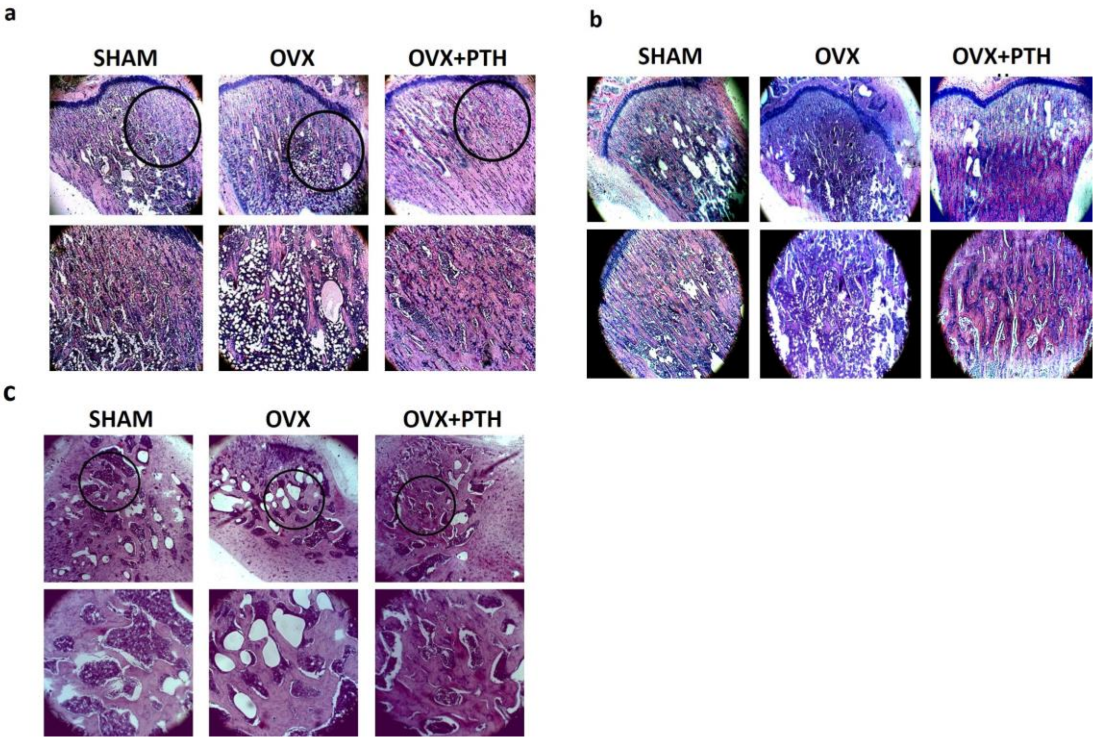

Figure 2: Prevention experiment reference measurements– the full set of bone marrow cellularity histology (Hematoxylin and Eosin stain) of SHAM, OVX and OVX+PTH, treated with PTH at 6-weeks post operation. Bone marrow cellularity histology of tibiae bones (a) and (b); and femurs bone (c). OVX revealed high number of adipocytes and less dense trabecular bone compared with the SHAM and PTH treated. Slides were visualized by Motic AE31 inverted microscope, magnification X4/X10.

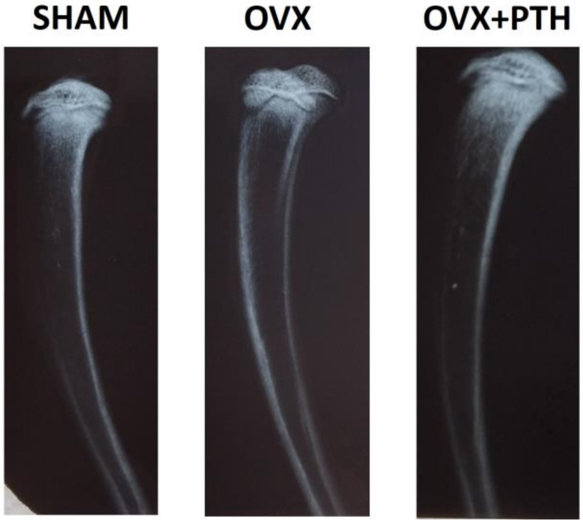

Figure 3: Prevention experiment reference measurements– representative radiographs of SHAM, OVX and OVX+PTH tibiae bones, treated with PTH at 6-weeks post operation. PTH treatment increased the trabecular bone when compared with the SHAM and the OVX controls. Slides were conducted with FAXITRON (a cabinet X-ray system, Hewlett-Packard).

1. 2. Therapeutic Treatment Experiment Additional Results

Figure 4 shows T_2 measurements of tibiae bones at the peripheral zone of bone marrow cavity, 11 weeks post operation. Although T_2 of OVX tibiae bones increased compare to the OVX+PTH and the SHAM femurs bones, it was so not significant ($P>0.05$).

T_1 and ADC measurements at the peripheral zone showed general increase of the OVX+PTH tibiae or femurs bones compare to the OVX groups (see Figure 5 and Figure 6 respectively), though it was not significant.

Unlike ADC measurement in the central zone of bone marrow cavity, T_2 measurement in the central zone showed no significant changes between the three groups (see Figure 7).

The full set of histological observations of the bone marrow and of SHAM, OVX and OVX+PTH bones, treated with PTH for 3 weeks at 8-weeks post operation (therapeutic experiment) are presented in Figure 8. Additional depictive microradiography images of tibiae bones are presented in Figure 9. The metaphysis of OVX tibiae bones is characterized by narrow trabeculli and low trabecular bone surface, while most of marrow spaces are filled with adipose tissue. In the PTH treated animals (PTH+OVX) pronounced hematopoietic marrow and wide trabeculii were present in metaphysis, however there is greater amount of adipocytes than SHAM rats.

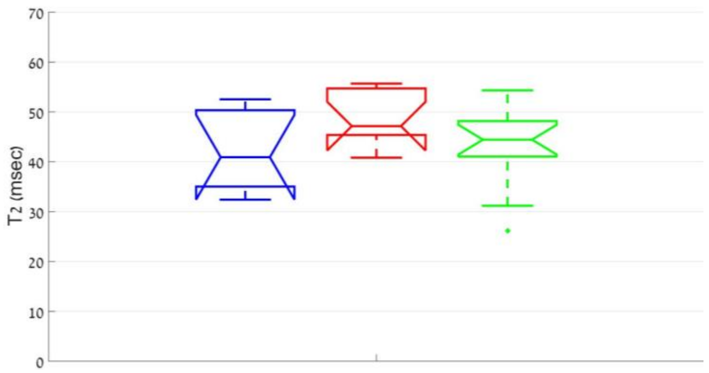

Figure 4: Therapeutic treatment experiment – T_2 measurements of tibiae bones at the peripheral zone of bone marrow cavity, 11 weeks post operation. No Significant increase for the tibiae OVX bones compare to the OVX+PTH and the SHAM femurs bones when measured in the peripheral zone.

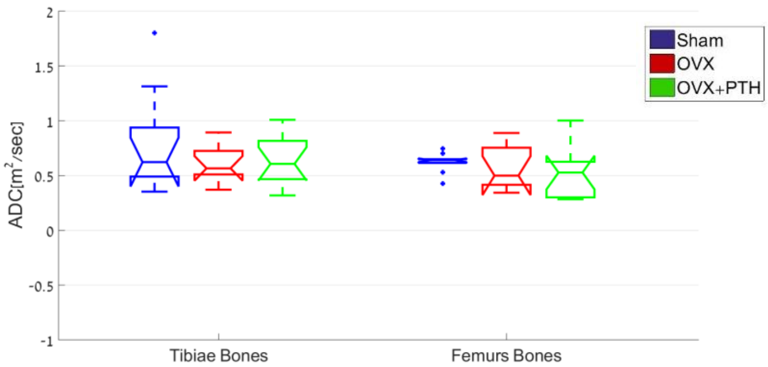

Figure 5: Therapeutic treatment experiment – ADC measurements of tibiae and femurs bones at the peripheral zone of bone marrow cavity, 11 weeks post operation. No Significant changes for the ADC value of the tibiae or femurs bones were observed when measured in the peripheral zone.

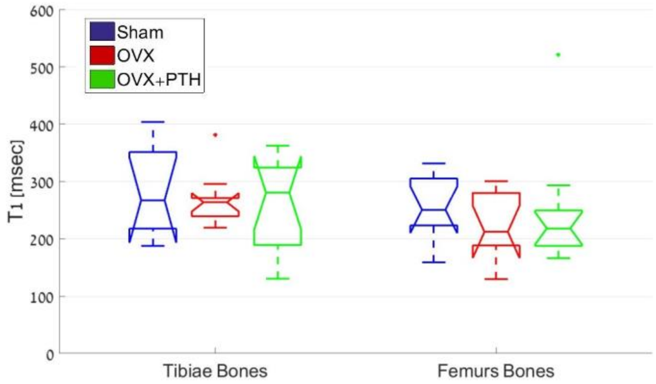

Figure 6: Therapeutic treatment experiment – T_1 measurements of tibiae and femurs bones at the peripheral zone of bone marrow cavity, when measured 11 weeks post ovariectomy. No Significant changes for the T_1 value of the tibiae or femurs bones were observed when measured in the peripheral zone.

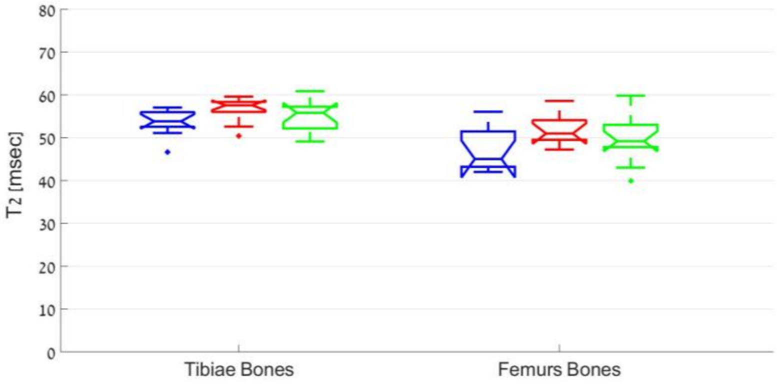

Figure 7: Therapeutic treatment experiment – T_2 measurements of tibiae and femurs bones at the central zone of bone marrow cavity, 11 weeks post operation. No Significant changes for the T_2 value of the tibiae or femurs bones were observed when measured in the central zone.

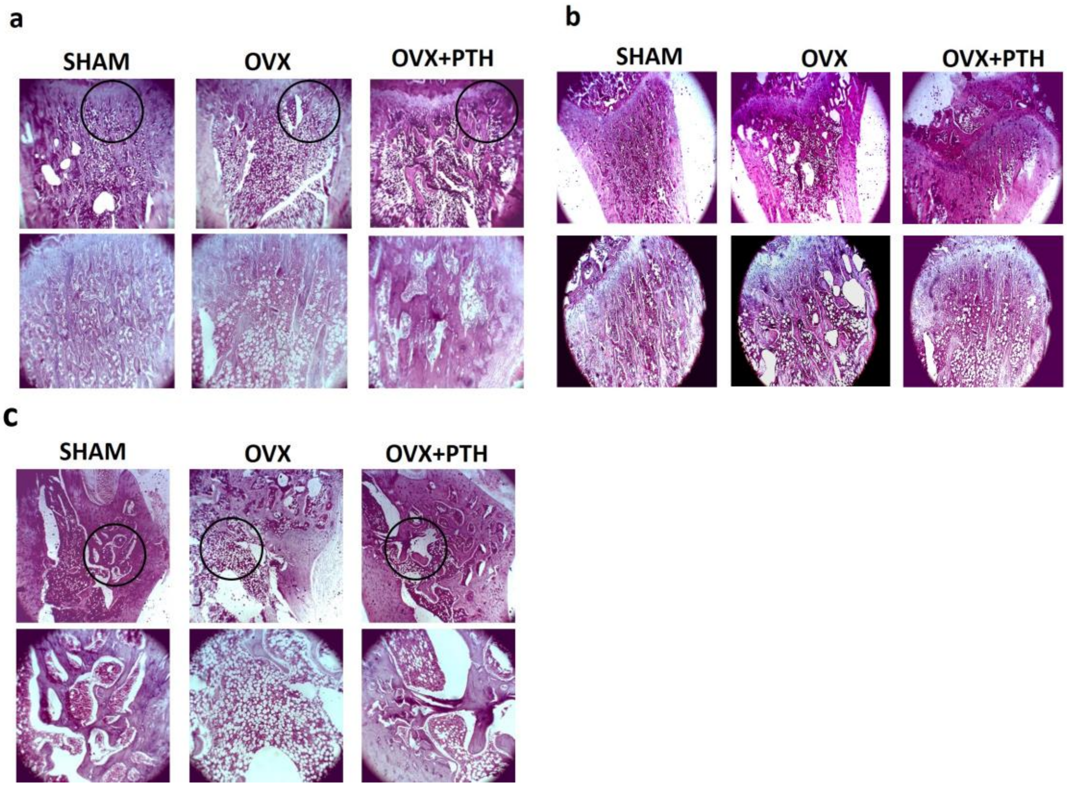

Figure 8: Therapeutic treatment experiment – full set of bone marrow cellularity histology (Hematoxylin and Eosin stain) of SHAM, OVX and OVX+PTH tibiae bones (a) and (b); and femurs bones (c) at 11-weeks post operation. OVX revealed greater amount of adipocytes compared with the SHAM and less denser bone than OVX+PTH bones. Slides were visualized by Motic AE31 inverted microscope, magnification X4/X10.

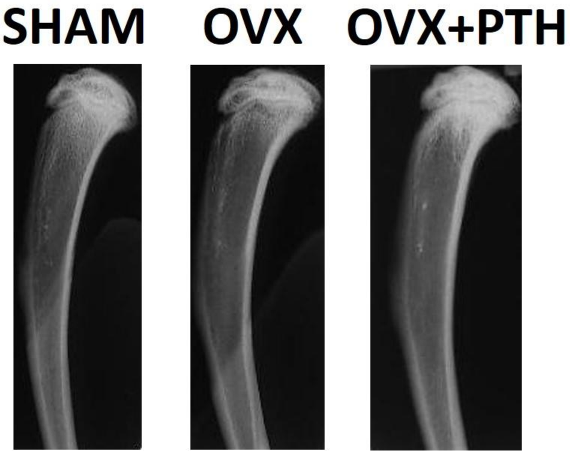

Figure 9: Therapeutic treatment experiment – representative radiographs of SHAM control, OVX and OVX treated with PTH tibiae bones at 11-weeks post operation. PTH treatment increased the trabecular bone compare with the SHAM and the OVX controls. Slides were conducted with FAXITRON (a cabinet X-ray system, Hewlett-Packard).
